# Cardiovascular Outcomes and Mortality Associated With Discontinuing Statins in Older Patients Receiving Polypharmacy

**DOI:** 10.1001/jamanetworkopen.2021.13186

**Published:** 2021-06-14

**Authors:** Federico Rea, Annalisa Biffi, Raffaella Ronco, Matteo Franchi, Simona Cammarota, Anna Citarella, Valeria Conti, Amelia Filippelli, Carmine Sellitto, Giovanni Corrao

**Affiliations:** 1 National Centre for Healthcare Research and Pharmacoepidemiology, University of Milano-Bicocca, Milan, Italy; 2 Laboratory of Healthcare Research and Pharmacoepidemiology, Unit of Biostatistics, Epidemiology, and Public Health, Department of Statistics and Quantitative Methods, University of Milano-Bicocca, Milan, Italy; 3 Department of Medicine, Surgery, and Dentistry, University of Salerno, Baronissi, Italy; 4 LinkHealth, Health Economics, Outcomes and Epidemiology, Naples, Italy

## Abstract

**Importance:**

Polypharmacy is a major health concern among older adults. While deprescribing may reduce inappropriate medicine use, its effect on clinical end points remains uncertain.

**Objective:**

To assess the clinical implications of discontinuing the use of statins while maintaining other drugs in a cohort of older patients receiving polypharmacy.

**Design, Setting, and Participants:**

This retrospective, population-based cohort study included the 29 047 residents in the Italian Lombardy region aged 65 years or older who were receiving uninterrupted treatment with statins, blood pressure–lowering, antidiabetic, and antiplatelet agents from October 1, 2013, until January 31, 2015, with follow-up through June 30, 2018. Data were collected using the health care utilization database of Lombardy region in Italy. Data analysis was conducted from March to November 2020.

**Exposures:**

Cohort members were followed up to identify those who discontinued statins. Among this group, those who maintained other therapies during the first 6 months after statin discontinuation were 1:1 propensity score matched with patients who discontinued neither statins nor other drugs.

**Main Outcome and Measures:**

The pairs of patients discontinuing and maintaining statins were followed up from the initial discontinuation until June 30, 2018, to estimate the hazard ratios (HRs) and 95% CIs for fatal and nonfatal outcomes associated with statin discontinuation.

**Results:**

The full cohort inclued 29 047 patients exposed to polypharmacy (mean [SD] age, 76.5 [6.5] years; 18 257 [62.9%] men). Of them, 5819 (20.0%) discontinued statins while maintaining other medications, and 4010 (68.9%) of them were matched with a comparator. In the discontinuing group, the mean (SD) age was 76.5 (6.4) years, 2405 (60.0%) were men, and 506 (12.6%) had Multisource Comorbidity Scores of 4 or 5. In the maintaining group, the mean (SD) age was 76.1 (6.3) years, 2474 (61.7%) were men, and 482 (12.0%) had multisource comorbidity scores of 4 or 5. Compared with the maintaining group, patients in the discontinuing group had increased risk of hospital admissions for heart failure (HR, 1.24; 95% CI, 1.07-1.43) and any cardiovascular outcome (HR, 1.14; 95% CI, 1.03-1.26), deaths from any cause (HR, 1.15; 95% CI, 1.02-1.30), and emergency admissions for any cause (HR, 1.12; 95% CI, 1.05-1.19).

**Conclusions and Relevance:**

In this study of patients receiving polypharmacy, discontinuing statins while maintaining other drug therapies was associated with an increase in the long-term risk of fatal and nonfatal cardiovascular outcomes.

## Key Points

**Question:**

What are the clinical implications of statin discontinuation in older patients receiving polypharmacy?

**Findings:**

In this population-based cohort study of 29 047 patients, there was evidence that discontinuing therapy with statins was associated with a significantly increased risk of hospital admission for heart failure and any cardiovascular outcome, death from any cause, and emergency admission for any cause.

**Meaning:**

The findings of this study suggest that discontinuing statins while maintaining other drug therapies may increase the long-term risk of fatal and nonfatal cardiovascular outcomes.

## Teaser

This population-based cohort study assesses the clinical implications of discontinuing the use of statins while maintaining other drugs in a cohort of older patients receiving polypharmacy.

## Introduction

Increasing life expectancy is doubtless one of the highest attainments of the 21st century.^1^ At the same time, an aging population has led to an increasing number of individuals affected by comorbidities.^2^ The latter, ie, the coexistence of multiple health problems, is practically a rule among older patients and is associated with lower quality of life.^3^ Almost unavoidably, comorbidities imply the need for polypharmacy.^4^ However, exposure to both multiple chronic diseases and polypharmacy may have negative clinical consequences. These include further pathological conditions, such as cognitive impairment, and adverse drug reactions due to drug-drug interactions.^5,6^ In this setting, increasing focus has been placed on deprescribing, ie, the process of gradually reducing or stopping drugs, to minimize polypharmacy and improve patients’ health outcomes.^7^ However, according to several randomized clinical trials,^8,9,10^ while deprescribing appears to reduce inappropriate medicine use, its effect on clinical end points, such as hospital admissions and survival, remains uncertain.

Statins are the most widely prescribed medication in the Western world, being a pivotal component in the primary and secondary prevention of cardiovascular (CV) diseases.^11,12,13,14,15^ However, for a variety of reasons, randomized clinical trials usually exclude patients with poor clinical conditions, which means that whether and to what extent statin administration plays a CV preventive role for frail patients, such as those with polypharmacy, is still unclear.^16^ Of course, this means that we cannot predict the implications of statin deprescribing in patients receiving polypharmacy.

The present study investigated a large cohort of patients aged 65 years or older exposed to polypharmacy. The primary objective was to assess clinical implications of discontinuing the use of statins while maintaining the use of other drugs. Controlling for sources of systematic uncertainty was of particular concern in this study.

## Methods

### Data Source

The data used for this study were retrieved from the health care utilization databases of Lombardy, a region of Italy that accounts for approximately 16% of the country’s population (>10 million individuals). In Italy, the whole population is covered by the National Health Service, and in Lombardy, this has been associated since 1997 with an automated system of databases to collect a variety of information, including an archive of hospitalizations (primary diagnosis, coexisting conditions, and procedures) and a database on outpatient drug prescriptions. Details of health care utilization databases of the Lombardy region and of their use in the field of CV diseases have been reported elsewhere.^17,18,19^

Specific diagnostic and therapeutic codes used for the current study are given in eTable 1 in the Supplement. Our manuscript was structured in accordance with Strengthening the Reporting of Observational Studies in Epidemiology (STROBE) reporting guideline for cohort studies. According to the rules from the Italian Medicines Agency, ^20^ retrospective studies using administrative databases do not require ethics committee protocol approval.

### Cohort Selection and Follow-up

#### Step 1

**Figure 1.**
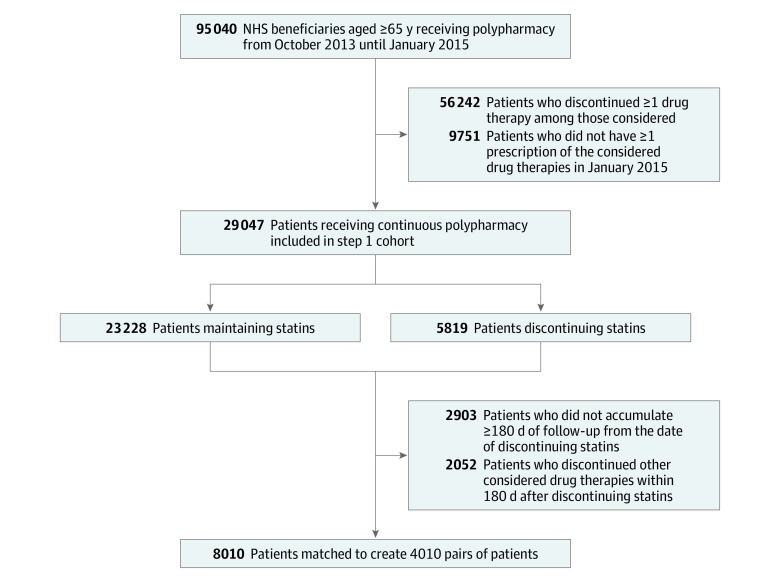
Flowchart of Selection of the Cohorts NHS indicates National Health System.

The study was designed according to a 2-step procedure, as described in Figure 1. The first step aimed to identify patients receiving polypharmacy and, among them, to identify those who discontinued statin therapy. With this aim, the nearly 2.3 million beneficiaries of the National Health Service who were aged at least 65 years and had their residence in Lombardy for at least 3 years during the pre–follow-up period (ie, October 1, 2013, to January 31, 2015) were identified. Among these, we found 95 040 patients who were receiving polypharmacy, ie, treatment with statins and blood pressure–lowering, antidiabetic, and antiplatelet agents, during the pre–follow-up period.

All prescriptions dispensed to these patients during the pre–follow-up period were identified, and the coverage duration of each prescription was calculated according to the defined daily dose metric. Consecutively refilled prescriptions were considered uninterrupted if the time between the end of one prescription and the beginning of the following prescription (or of censoring) was 90 days or shorter. Discontinuation was assumed otherwise.

To be included in the step 1 cohort, a patient had to have continuous treatment with all 4 drug treatments during the pre–follow-up period, ie, a patient was excluded if they had at least 1 episode of discontinuation with any lipid-lowering, blood pressure–lowering, antidiabetic, or antiplatelet agents. Another requirement for entering the step 1 cohort was that January 2015 had to be covered by at least 1 prescription of all the considered drug therapies.

The 29 047 patients who met these criteria were included in the step 1 cohort, whose members accumulated person-years (PYs) of follow-up from February 1, 2015, until death from any cause, emigration, or the step 1 end point (ie, June 30, 2017). The end point was established as the occurrence of statin discontinuation. The latter occurred when a given prescription was not renewed within 90 days from the end of its coverage during follow-up, and the last date of coverage before interrupting was assumed as the end point occurrence (eFigure 1 in the Supplement).

#### Step 2

The second step aimed to compare the incidence of several clinical outcomes among patients who discontinued statins while maintaining the other drug therapies with patients who did not discontinue either statins or other drug therapies. With this aim, for each of the 5819 step 1 cohort members who experienced the end point, a new follow-up (step 2 follow-up) started at the date of statin discontinuation. We excluded patients who did not accumulate at least 180 days of step 2 follow-up as well as those who discontinued another drug therapy (ie, blood pressure–lowering, antidiabetic, or antiplatelet agents) during this period. The remaining 4023 patients represented the portion of step 2 cohort members who discontinued statins while maintaining other treatments and were therefore labeled as exposed to statin deprescribing.

A step 1 cohort member was eligible to be included as comparator of a given member of the discontinuing group if, at the date of the latter’s statin discontinuation, the potential comparator (1) had not yet experienced any statin interruption (ie, a density incidence was used for selecting comparators) and (2) accumulated at least 180 days of follow-up, during which they experienced uninterrupted treatment with statins as well as with the other drug therapies considered (eFigure 2 in the Supplement).

Given that persistence with statins was likely affected by clinical profile and other relevant characteristics, a 1:1 propensity score (PS) matching design was adopted.^21^ Exposure PS was derived by regressing selected baseline covariates toward the exposure of interest (ie, discontinuing vs maintaining therapy with statins) with a logistic regression model. Covariates were sex; age on February 1, 2015; comorbidities (ie, cancer, cerebrovascular disease, ischemic heart disease, heart failure, respiratory disease, kidney disease, and liver disease) and adherence to statins, blood pressure–lowering, antidiabetic, and antiplatelet agents during the step 1 follow-up according to the proportion-of-days-covered metric.^22^ In addition, we considered the Multisource Comorbidity Score (MCS), which is a new index of patients’ clinical status based on 34 CV and non-CV conditions (eg, heart failure, arrhythmia, cancer) derived from inpatient diagnostic information and outpatient drug prescriptions and weighted according to their strength with mortality.^23,24^ Members in the statin discontinuing and maintaining groups were then 1:1 matched on their PS using a nearest neighbor matching algorithm without replacement with a caliper of 0.01.^25^

Pairs in the statin discontinuing and maintaining groups accumulated PYs of follow-up from 180 days after the discontinuation date of the exposed member (ie, the starting point for patients in the maintaining group was set at 180 days after the discontinuation date of the paired discontinuing patient) until the clinical outcome of interest or censoring (emigration or end of data availability, ie, June 30, 2018) (eFigure 3 in the Supplement). Several clinical outcomes were considered separately. Five separate outcomes included hospital admission for cerebrovascular disease, heart failure, or ischemic heart disease; emergency department admission for any cause or neurologic disorders; and death for any cause. Two composite outcomes included hospital admission for CV causes (ie, cerebrovascular disease, heart failure, or ischemic heart disease) and hospital admission for these causes or death for any cause.

### Statistical Analysis

#### Data Analyses

The discontinuation rate was expressed as the ratio between the number of discontinuation episodes and PYs accumulated during the step 1 follow-up. With the aim of verifying between-group differences in baseline characteristics, the standardized difference was calculated for each measured covariate before and after PS matching of step 2 cohort members from the discontinuing and maintaining groups. A Cox proportion hazard model was used for estimating hazard ratios (HRs) and corresponding 95% CIs of the outcomes of interest associated with exposure to statin discontinuation. Analyses were also performed according to strata of sex, age (on February 1, 2015), previous CV events (ie, according to whether statins were prescribed for primary or secondary prevention), and clinical profile (ie, according to categories of MCS).

#### Sensitivity Analyses

After having recognized some potential weaknesses of the design and analysis as previously described, we verified the robustness of the main findings by adopting an alternative second-step design. Weakness first concerned the implicit assumption of the intention-to-treat principle for defining exposure to statin discontinuation and maintaining other drug therapies. Given that patients who discontinued statins may resume them and that patients who maintained use of statins during the first 180 days of follow-up may interrupt them afterward, exposure misclassification may affect the main results. For this reason, a Cox proportional hazard model including the exposure to statins during the entire follow-up as a time-dependent covariate was fitted.

In addition, although the other treatments (ie, blood pressure–lowering, antidiabetic, and antiplatelet agents) were maintained during the first 180 days of follow-up, their use may have been interrupted later. Because the aim of our study was to assess the clinical implications of discontinuing the use of statins while maintaining the use of other drugs, we decided to censor the follow-up of cohort members when another cotreatment was interrupted. However, it should be considered that the reasons for discontinuing may be associated with the outcome onset, so generating estimates were biased by informative censoring. This is why an inverse probability of censoring weights (IPCW) approach was adopted. This method may be considered analogous to the previously described PS method, considering that both methods aimed to identify characteristics able to stand in for confounders. The difference consists in the fact that while PS includes time-fixed covariates, IPCW refers to time-varying covariates. In brief, the design implies that information was censored when the use of other treatments (ie, blood pressure–lowering, antidiabetic, and antiplatelet agents) was interrupted for the first time during follow-up. The IPCW method consists of assigning each patient a weight inversely proportional to the probability of censoring, conditional to covariates measured until censoring, stratifying for step 2 cohort members in both the discontinuing and maintaining groups. Weights were generated by fitting a time-dependent Cox regression model to estimate the probability of changing the status of other cotreatments. Potential characteristics for estimating censoring were the possible causes of hospital discharge experienced by and drugs prescribed to step 2 cohort members from 180 days after entering into step 2 cohort until censoring occurred. The 200 most prognostic covariates were selected. The censoring weights were then stabilized by the probability of censoring conditional on the treatment with statins received.^26^ A Cox proportional hazard model was then fitted for estimating HRs and corresponding 95% CIs of the outcomes of interest associated with exposure to statin discontinuation, weighting each patient with the IPCW estimated as previously described.

Finally, we speculated that the association between statin discontinuation and the risk of health outcomes we studied might be due to unmeasured confounding by health-seeking behaviors of healthier patients.^27^ To investigate this issue, we repeated all the previously described analyses by replacing statins with a medication that we expected to be independent from the outcomes we studied (ie, a negative exposure). With this aim, we identified a new cohort of individuals using proton pump inhibitors (as well as blood pressure–lowering, antidiabetic, and antiplatelet agents, as in the main analysis). The association of discontinuing proton pump inhibitors while maintaining the other drug therapies with survival was then investigated according to the previously described analyses. Given that the long-term use of proton pump inhibitors is unlikely to be associated with better survival, we did not expect any association between discontinuation of this drug therapy and the risk of the considered outcome.

All analyses were performed using the SAS version 9.4 (SAS Institute). For all hypotheses tested, a 2-tailed *P* < .05 was considered statistically significant.

## Results

### Identifying Patients Receiving Polypharmacy Who Discontinued Statins

The 29 047 patients exposed to polypharmacy showed a high adherence to treatment in the pre–follow-up period (eTable 2 in the Supplement). The cohort members had mean (SD) age of 76.5 (6.5) years, 18 257 (62.9%) were men, almost 1 in 5 had experienced ischemic heart disease (5735 [19.7%]), while 1 in 12 had cerebrovascular disease (2280 [7.9%]), heart failure (2299 [7.9%]), and/or respiratory disease (2354 [8.1%]) (Table 1). On the whole, two-thirds of the included patients had any comorbidity, and more than 1 in 10 had a severe clinical profile (ie, MCS score ≥4; 3405 [11.7%])).

**Table 1.** Baseline Characteristics of Step 1 Cohort

Characteristics	Patients, No. (%) (N = 29 047)
Age, mean (SD), y	76.5 (6.5)
Men	18 257 (62.9)
Women	10 790 (37.1)
Comorbidities	
Cerebrovascular disease	2280 (7.9)
Ischemic heart disease	5735 (19.7)
Heart failure	2299 (7.9)
Respiratory disease	2354 (8.1)
Kidney disease	1543 (5.3)
Liver disease	149 (0.5)
Cancer	1404 (4.8)
Multisource comorbidity score	
1	9765 (33.6)
2	10 444 (36.0)
3	5433 (18.7)
4	2106 (7.3)
5	1299 (4.5)

The step 1 cohort accumulated 70 230 PYs of follow-up, approximately 2.4 years per patient. During this period, 9204 patients (31.7%) discontinued statins (the step 1 outcome), with an incidence of 13.1 events per every 100 person-years. Among these 9204 patients, 5819 (63.2%) discontinued statins before interrupting another drug therapy.

### Assessing Clinical Consequence of Discontinuing Statins

Among the 5819 patients who discontinued statins (as first treatment), 4203 patients were eligible for matching. They were older, were more likely to be women, and were less likely to have heart failure than the 18 273 patients who maintained lipid-lowering therapy (mean [SD] age, 76.5 [6.5] years vs 75.3 [6.3] years; 2507 [59.7%] men vs 11 807 [64.6%] men; 340 [8.1%] vs 1553 [8.5%] with heart failure ) (Table 2). After matching, the 4010 1:1 PS matched pairs had better between-group balancing for all considered characteristics, including antihypertensive drug classes used in heart failure (eTable 3 in the Supplement).

**Table 2.** Comparing Selected Characteristics of Cohort Members Who Discontinued and Maintained Therapy With Statins

Characteristic	Raw comparison			Propensity score matching design		
	Patients discontinuing, No. (%) (n = 4203)	Patients maintaining, No. (%) (n = 18 273)	Standardized difference	Patients discontinuing, No. (%) (n = 4010)	Patients maintaining, No. (%) (n = 4010)	Standardized difference
Age, mean (SD), y	76.5 (6.5)	75.3 (6.3)	0.188	76.5 (6.4)	76.1 (6.3)	0.063
Men	2507 (59.7)	11 807 (64.6)	−0.103	2405 (60.0)	2474 (61.7)	−0.035
Women	1696 (40.3)	6466 (35.4)		1605 (40.0)	1536 (38.3)	
Comorbidities						
Cerebrovascular disease	373 (8.9)	1622 (8.8)	0.000	358 (8.9)	323 (8.1)	0.031
Ischemic heart disease	821 (19.5)	4329 (23.7)	−0.101	775 (19.3)	778 (19.4)	−0.002
Heart failure	340 (8.1)	1553 (8.5)	−0.105	320 (8.0)	293 (7.3)	0.025
Respiratory disease	390 (9.3)	1585 (8.7)	0.021	368 (9.2)	320 (8.0)	0.043
Kidney disease	240 (5.7)	1012 (5.5)	0.007	212 (5.3)	190 (4.7)	0.025
Liver disease	21 (0.5)	106 (0.6)	−0.011	20 (0.5)	28 (0.7)	−0.026
Cancer	216 (5.1)	967 (5.3)	−0.007	202 (5.0)	198 (4.9)	0.005
Multisource comorbidity score						
1	1002 (23.8)	4522 (24.8)	−0.021	965 (24.1)	1008 (25.1)	−0.025
2	1773 (42.2)	7618 (41.7)	0.010	1697 (42.3)	1665 (41.5)	0.016
3	887 (21.1)	3890 (21.3)	−0.005	842 (21.0)	855 (21.3)	−0.008
4	328 (7.8)	1417 (7.8)	0.002	312 (7.8)	287 (7.2)	0.024
5	213 (5.1)	826 (4.5)	0.026	194 (4.8)	195 (4.9)	−0.001

After a mean (SD) follow-up of 20.6 (10.0) months and 20.4 (10.1) months for patients who discontinued and maintained statins, respectively, these patients experienced hospital admissions for cerebrovascular disease (235 events vs 208 events; incidence rate, 35.8 per 1000 PY vs 31.2 per 1000 PY), for heart failure (408 events vs 337 events; 64.0 per 1000 PY vs 51.5 per 1000 PY), and for ischemic heart disease (439 events vs 413 events; 69.7 per 1000 PY vs 64.6 per 1000 PY). They experienced admissions in emergency department for any cause (2209 events vs 2055 events; 506.2 per 1000 PY vs 449.8 per 1000 PY) and for neurologic disorders (346 events vs 330 events; 53.4 per 1000 PY and 50.4 per 1000 PY) and deaths for any-cause (528 events vs 463 events; 77.5 per 1000 PY vs 67.4 per 1000 PY).

**Figure 2.**
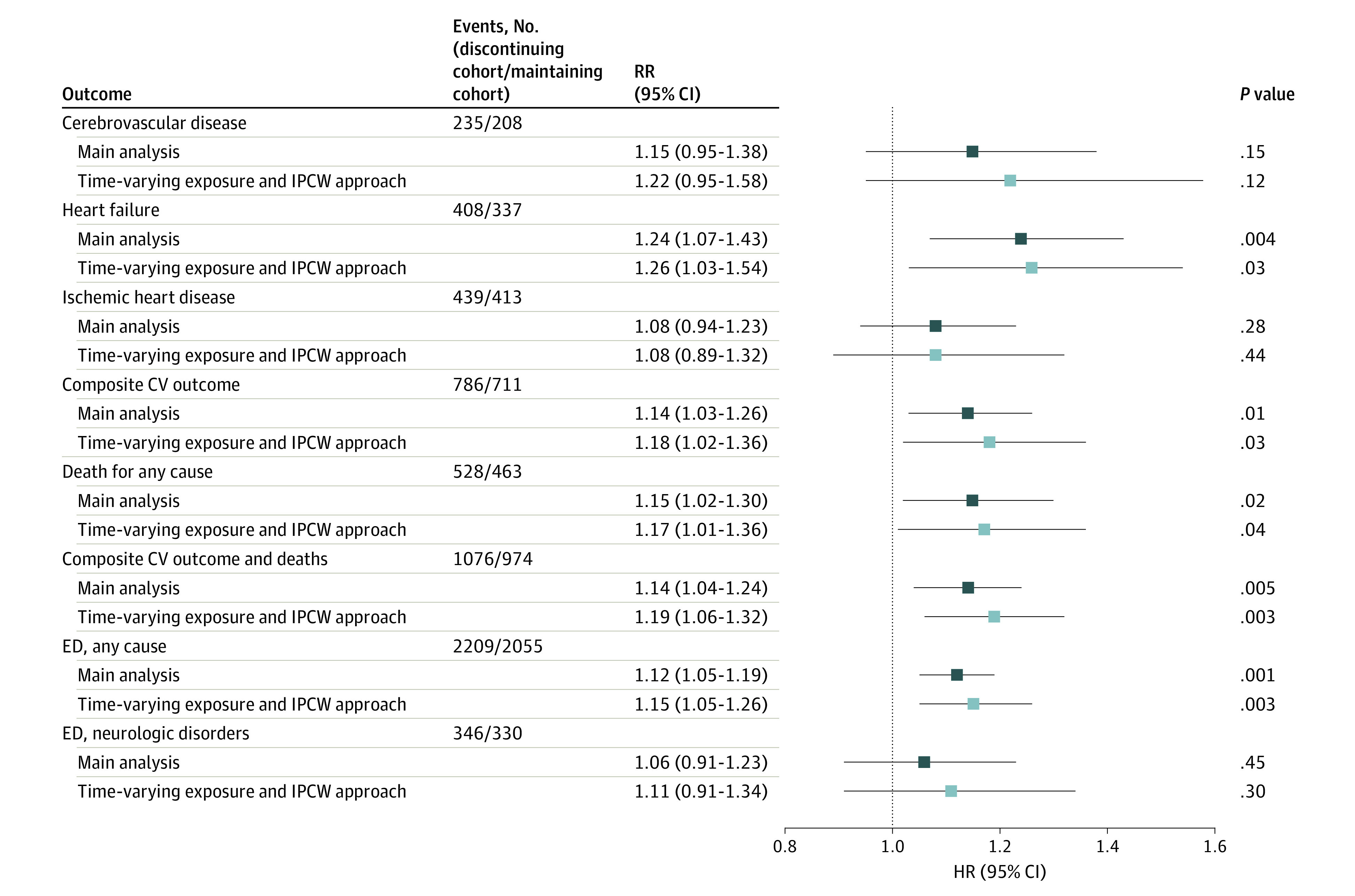
Association of Discontinuing Statin Therapy With Risk of Hospital Admission for Cardiovascular Outcomes, All-Cause Mortality, and Admission in Emergency Department Estimates from intention-to-treat time fixed and as-treated inverse probability censoring weights (IPCW) designs are reported. ED indicates emergency department; HR, hazard ratio.

Forest plots representing the association between discontinuation with statins and the risk of these outcomes as well as of some aggregate outcome are shown in Figure 2. With the exception of hospital admission for cerebrovascular disease and ischemic heart disease and emergency admission for neurologic disorders, there was evidence that discontinuing therapy with statins increased the risk of all the other considered outcomes (eg, risk of hospitalization for heart failure: HR, 1.24; 95% CI, 1.07-1.43; any cardiovascular outcome: HR, 1.14; 95% CI, 1.03-1.26; death from any cause: HR, 1.15; 95% CI, 1.02-1.30; emergency admissions for any cause: HR, 1.12; 95% CI, 1.05-1.19). Estimates were consistent whether they were obtained from the intention-to-treat time-fixed approach, or through an as-treated/IPCW design.

Stratified analyses (Table 3) did not show evidence that the effect of statin discontinuation was significantly heterogeneous between categories of sex, age, and clinical profile, nor according to whether discontinuation concerned a setting of primary or secondary prevention of CV events. Finally, according to negative exposure analysis, there was no evidence that discontinuing proton pump inhibitors affected mortality (HR, 1.08; 95% CI, 0.95-1.22).

**Table 3.** Association of Hospital Admission for Cardiovascular Outcomes, All-Cause Mortality, and Admission in Emergency Department, According to Sex, Age Classes, Clinical Profile, and Preventive Setting

Characteristic	HR (95% CI)		
	Combined CV	All-cause mortality	Emergency department
Sex			
Men	1.16 (1.02 to 1.31)	1.21 (1.03 to 1.42)	1.11 (1.03 to 1.20)
Women	1.10 (0.93 to 1.31)	1.06 (0.87 to 1.30)	1.12 (1.02 to 1.23)
*P* value	.67	.31	.93
Age, y			
65-75	1.13 (0.97 to 1.32)	1.24 (0.99 to 1.57)	1.11 (1.01 to 1.22)
76-85	1.14 (0.98 to 1.33)	1.05 (0.88 to 1.25)	1.10 (1.01 to 1.20)
>85	1.03 (0.77 to 1.36)	1.07 (0.82 to 1.40)	1.09 (0.91 to 1.30)
*P* value	.75	.38	.80
MCS			
1	0.90 (0.69 to 1.18)	0.99 (0.70 to 1.40)	1.12 (0.98 to 1.28)
2	1.31 (1.11 to 1.55)	1.31 (1.05 to 1.63)	1.22 (1.11 to 1.35)
≥3	1.02 (0.84 to 1.25)	1.10 (0.87 to 1.40)	0.94 (0.83 to 1.07)
*P* value	.69	.69	.18
Preventive setting			
Secondary	1.12 (0.96 to 1.31)	1.16 (0.95 to 1.41)	1.05 (0.94 to 1.16)
Primary	1.14 (1.00 to 1.30)	1.14 (0.97 to 1.34)	1.14 (1.06 to 1.23)
*P* value	.90	.92	.18

Abbreviations: CV, cardiovascular; HR, hazard ratio; MCS, Multisource Comorbidity Score.

## Discussion

In our large, population-based, observational investigation we found that among patients on polypharmacy, those who discontinued statins while maintaining other drug therapies were at a higher long-term risk of CV hospital admission, all-cause emergency admission, and all-cause mortality compared with those who maintained all drug therapies, including statins. The augmented risks were not trivial because, compared with maintaining all drugs, discontinuation of statins only was associated with risk excesses ranging from 12% (all-cause emergency admission) to 24% (heart failure). On the other hand, the simplification of the polypharmacy burden in these patients did not generate a significant reduction in access to the emergency department for neurological causes, considered a proxy for the onset of episodes of delirium. Finally, likely because of the limited power, more uncertain findings were obtained when the association was investigated among the oldest patients and those with a worse clinical profile.

Through a vast array of studies, it has been consistently shown that elevated levels of low-density lipoprotein cholesterol (LDL-C) increase the risk of developing atherosclerotic plaques and subsequent vascular disease.^28^ Several randomized clinical trials of treatment with lipid-lowering agents have shown that lowering LDL-C levels reduces the risk of atherosclerotic CV events in the future.^29,30,31^ On the other hand, it has been shown that the relapse rate of dyslipidemia (LDL-C rebounding to levels greater than 100 mg/dL [to convert to micromoles per liter, multiply by 0.0259]) was higher among patients receiving rosuvastatin who, having reached LDL-C levels lower than 100 mg/dL, were randomly assigned to discontinue statin treatment while others were assigned to maintain therapy.^32^ Finally, convincing evidence that statin discontinuation leads to adverse outcomes in high-risk patients, ie, those with acute coronary syndrome, those with ischemic stroke, or recipients of vascular surgery, was supplied from several studies.^33,34,35,36,37^ All this evidence taken together agrees with our conclusion of unfavorable consequences of discontinuing lipid-lowering treatment.

The main weakness of our study is that we do not know why statins were discontinued, but we can speculate on this issue. First, there may have been onset of adverse effects. However, more than 30 years of clinical investigation have shown that statins exhibit few serious adverse effects. Statin-induced newly diagnosed diabetes; muscle symptoms interfering with treatment compliance; myopathy, including rhabdomyolysis; and severe liver toxic effects occur in approximately 0.2%, less than 1%, less than 0.1%, and approximately 0.001%, respectively, of patients receiving statins per year.^38^ This means that although a small proportion of patients may have interrupted therapy because of the onset of an adverse effect, it is unlikely that safety concerns explain the high discontinuation incidence observed in our study (approximately 13 episodes of interruption every 100 PY). A second reason may be linked with deprescribing, ie, the process of gradually stopping drugs in patients exposed to polypharmacy.^7^ The choice of including patients who discontinued only statins while keeping other treatments went toward this direction, ie, simulating what happens when a drug (statin) is omitted to simplify the therapeutic scheme. The approach, of course, risks introducing several biases, mostly concerning confounding. For example, it is reasonable to imagine that deprescribing mostly affects patients with a lipid profile that is not so severe, justifying interruption of lipid-lowering therapy. However, it should be emphasized that if this were true, patients who discontinued would be characterized by a lower risk profile at baseline, and a reduction in CV risk and mortality should therefore be observed. Conversely, the choice of interrupting therapy with statins may be due to the sudden worsening of the clinical profile, which in turn may cause the observed risk excesses. Although possible, it is unlikely that this affected our findings for at least 2 reasons. First, it is unclear why the sudden worsening of clinical profile would affect the discontinuation of statins only, rather than other drugs. Second, we attempted to account for changing clinical profile by using all available information on the use of health services, identifying health services associated with the discontinuation of treatment with statins and other treatments, assuming that the latter are proxies for the complex of causes generating discontinuation with statins and other treatments, and correcting the estimates with a model that included these factors (ie, with a IPCW model^26^). Finally, the observed favorable effects of maintaining lipid-lowering treatment may be because of unmeasured confounding by health-seeking behaviors of healthier patients, ie, the so-called healthier user effect.^27^ If this were true, increased mortality should be observed for medication classes whose discontinuation is not anticipated to causally affect mortality. However, given that we did not observe any association between discontinuing therapy with pump inhibitors and mortality, this possibility seems unlikely.

Our study has several strengths. First, the investigation was based on a large unselected population, which was made possible because the cost-free health care system in Italy includes virtually all citizens.^39,40,41^ Second, the drug prescription database provides accurate data because pharmacists are required to report dispensations in detail to obtain reimbursement, and incorrect reports have legal consequences.^42^ Third, the choice of active comparison of patients receiving polypharmacy with the same indications at baseline, and even with the same level of adherence to and persistence of 4 active drug therapies, reduces the potential for confounding.^43^ Fourth, the inclusion of all-cause mortality as the main outcome avoided any uncertainty regarding diagnostic accuracy. Fifth, the consistency of estimates provided by sensitivity analyses indicates the robustness of our findings.

### Limitations

Our study has limitations. One, adherence to treatment was derived from drug prescriptions, ie, a widely used method to estimate adherence to treatment in large populations.^44^ However, this assumes that the proportion of days covered by a prescription corresponds with the proportion of days of drug use.^39^ Second, exposure misclassification may also occur because treatments delivered by private services and out-of-pocket payments for health care services were not tracked in our database.^45^

Third, because the allocation of discontinuing and/or deprescribing was not randomized, the results may be affected by confounding. That is, the observed increased risk of the considered clinical outcomes associated with discontinuing and/or deprescribing lipid-lowering medications might have been generated by factors accompanying but different from discontinuing and/or deprescribing. In fact, the measures described previously to account for between-group imbalance for unmeasured factors through proxies of severity of hyperlipidemia and clinical profile do not entirely eliminate the problem of confounding. For example, because discontinuing may be a surrogate for overall health-seeking behavior, patients who discontinued statins might also have less closely followed healthy lifestyle advice and less effectively treated other CV risk factors. However, as discontinuation regarded statins only, while blood pressure, antidiabetic, and antiplatelet agents were kept, this explanation of the results, although in principle possible, must be considered unlikely.

## Conclusions

In this study of patients aged 65 years or older who were exposed to polypharmacy, discontinuing therapy with statins while maintaining blood pressure–lowering, antidiabetic, and antiplatelet drug therapies was associated with an increased risk of fatal and nonfatal CV outcomes. This occurred in younger and older patients, men and women, patients with mild or severe clinical profiles, and irrespective of whether statins were prescribed in as primary or secondary CV prevention.

## Notes

Supplement. **eTable 1. **Diagnostic and Drug Codes Used for Study **eTable 2. **Proportion of Days Covered by Drug Treatments During the Pre–Follow-up Period **eTable 3. **Comparing Selected Characteristics of Cohort Members Who Discontinued and Maintained Therapy With Statins **eFigure 1.** Representation of First-Step Procedure Aimed to Identify Patients Who Discontinued Statin Therapy **eFigure 2.** Representation of Second-Step Procedure Aimed to Identify a Comparator for Each Patient Who Discontinued Statin Therapy **eFigure 3.** Representation of Step-2 Follow-up Aimed to Assess Clinical Consequence of Discontinuing Statins
