# Supplementary material for: Cardiovascular Outcomes and Mortality Associated With Discontinuing Statins in Older Patients Receiving Polypharmacy
Source: JAMA Netw Open. 2021 Jun 14;4(6):e2113186. doi: 10.1001/jamanetworkopen.2021.13186 (PMC8204202; doi:10.1001/jamanetworkopen.2021.13186)
Supplement: Supplement. — eTable 1. Diagnostic and Drug Codes Used for Study eTable 2. Proportion of Days Covered by Drug Treatments During the Pre–Follow-up Period eTable 3. Comparing Selected Characteristics of Cohort Members Who Discontinued and Maintained Therapy With Statins eFigure 1. Representation of First-Step Procedure Aimed to Identify Patients Who Discontinued Statin Therapy eFigure 2. Representation of Second-Step Procedure Aimed to Identify a Comparator for Each Patient Who Discontinued Statin Therapy eFigure 3. Representation of Step-2 Follow-up Aimed to Assess Clinical Consequence of Discontinuing Statins [file jamanetwopen-e2113186-s001.pdf]

Supplemental Online Content

Rea F, Biffi A, Ronco R, et al. Cardiovascular outcomes and mortality associated with discontinuing statins in older patients receiving polypharmacy. *JAMA Netw Open.* 2021;4(6):e2113186. doi:10.1001/jamanetworkopen.2021.13186

eTable 1. Diagnostic and Drug Codes Used for Study

eTable 2. Proportion of Days Covered by Drug Treatments During the Pre–Follow-up Period

eTable 3. Comparing Selected Characteristics of Cohort Members Who Discontinued and Maintained Therapy With Statins

eFigure 1. Representation of First-Step Procedure Aimed to Identify Patients Who Discontinued Statin Therapy

eFigure 2. Representation of Second-Step Procedure Aimed to Identify a Comparator for Each Patient Who Discontinued Statin Therapy

eFigure 3. Representation of Step-2 Follow-up Aimed to Assess Clinical Consequence of Discontinuing Statins

This supplemental material has been provided by the authors to give readers additional information about their work.

© 2021 Rea F et al. *JAMA Network Open.*

eTable 1. Diagnostic and Drug Codes Used for Study

**Diseases / conditions †**	**Codes**
Heart failure	428.x, 398.91, 402.01, 402.11, 402.91, 404.01, 404.03, 404.11, 404.13, 404.91, 404.93
Cerebrovascular disease	430.x-438.x
Ischemic heart disease	410.x-412.x, 414.x
Respiratory disease	460.x – 519.x
Liver disease	571.x, 573.8, 573.9
Renal disease	580.x – 589.x
Malignancies	140.x-208.x
**Drugs §**	
Antidiabetic agents	A10
Antithrombotic agents	B01AC
Statins	C10AA
Antihypertensive agents	C02, C03, C07, C08, C09

† According to the ICD-9-CM (International Classification of Disease, 9th Revision) system

§ According to the ATC (Anatomical-Therapeutic-Chemical) classification system

© 2021 Rea F et al. *JAMA Network Open.*

eTable 2. Proportion of Days Covered by Drug Treatments During the Pre–Follow-up Period

Drug therapy	Mean (SD)
Statins	0.79 (0.18)
Antihypertensive agents	0.93 (0.13)
Antidiabetic agents	0.83 (0.18)
Antiplatelet agents	0.80 (0.14)

© 2021 Rea F et al. *JAMA Network Open.*

eTable 3. Comparing Selected Characteristics of Cohort Members Who Discontinued and Maintained Therapy With Statins

Antihypertensive drug class	Discontinuing (n =4,010)	Keeping (n =4,010)	Standardized differences
Renin-angiotensin-system (RAS)-acting agents	3,718 (92.7%)	3,731 (93.0%)	-0.013
Beta-blockers	2,845 (71.0%)	2,864 (71.4%)	-0.010
Mineralocorticoid Receptor Antagonists	519 (12.9%)	488 (12.2%)	0.023

© 2021 Rea F et al. *JAMA Network Open.*

eFigure 1. Representation of First-Step Procedure Aimed to Identify Patients Who Discontinued Statin Therapy

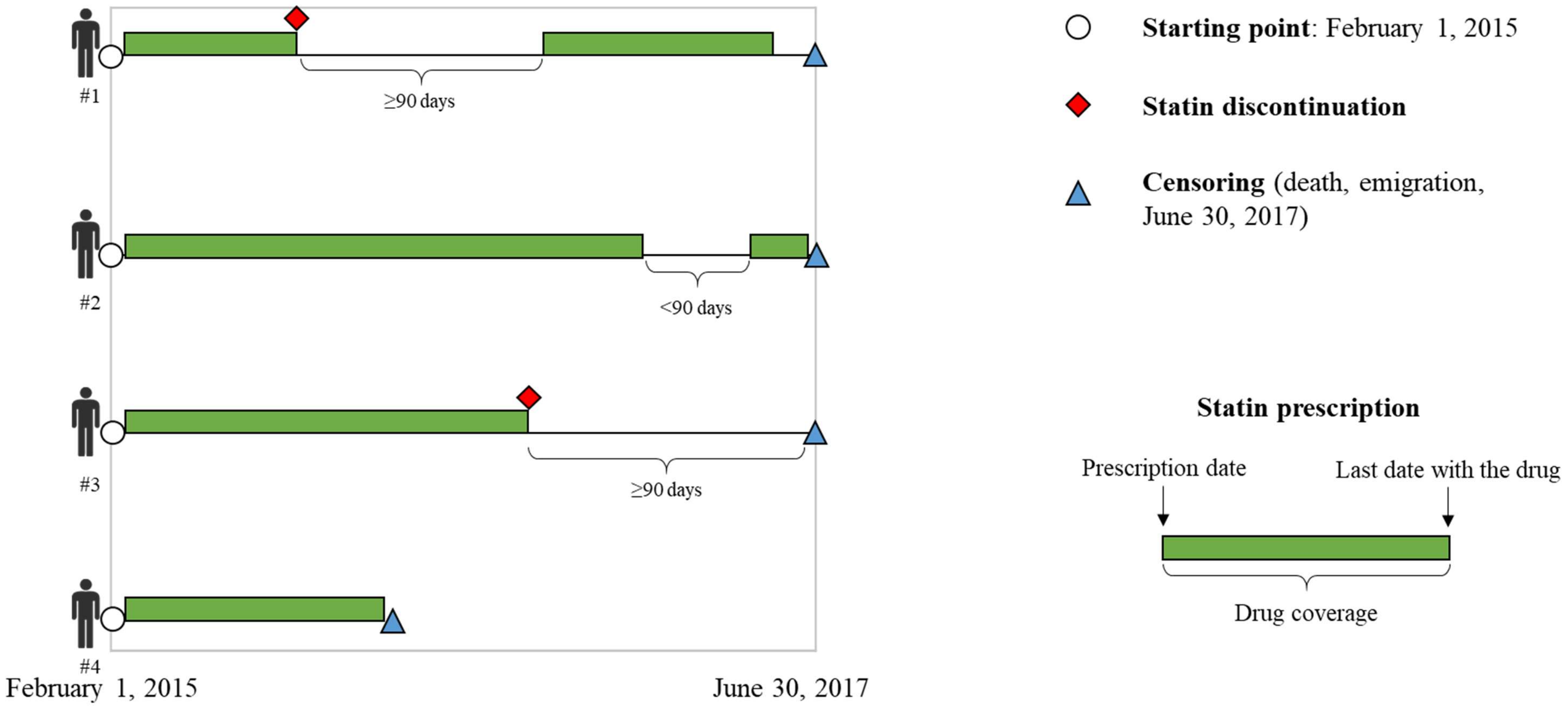

Patients were followed from February 1, 2015 until the occurrence of statin discontinuation or censoring (the earliest among death from any cause, emigration or end of step-1 follow-up). With the aim of having available at least one year of step-2 follow-up, we set the end-point of step-1 follow-up at June 30, 2017 (i.e., if we had prolonged the step-1 follow-up beyond, some patients could not have been observed for long enough to appreciate the effects of stopping statins in step-2 follow-up).

© 2021 Rea F et al. *JAMA Network Open.*

Statin prescriptions were considered uninterrupted if the time-span between the end of the coverage of one prescription and the beginning of the following one was less than 90 days; if the between-prescription timespan was longer, treatment discontinuation was assumed. Therefore, patients #1 and #3 discontinued statin therapy according to this definition (albeit patient #1 renew the drug therapy later). Conversely, patient #2 who spent some days in which the drug was not available was not classified as statin discontinuer (because this period was less than 90 days).

The last day in which the drug was available (before the 90-day period without drug) was defined as the discontinuation date.

© 2021 Rea F et al. *JAMA Network Open.*

eFigure 2. Representation of Second-Step Procedure Aimed to Identify a Comparator for Each Patient Who Discontinued Statin Therapy

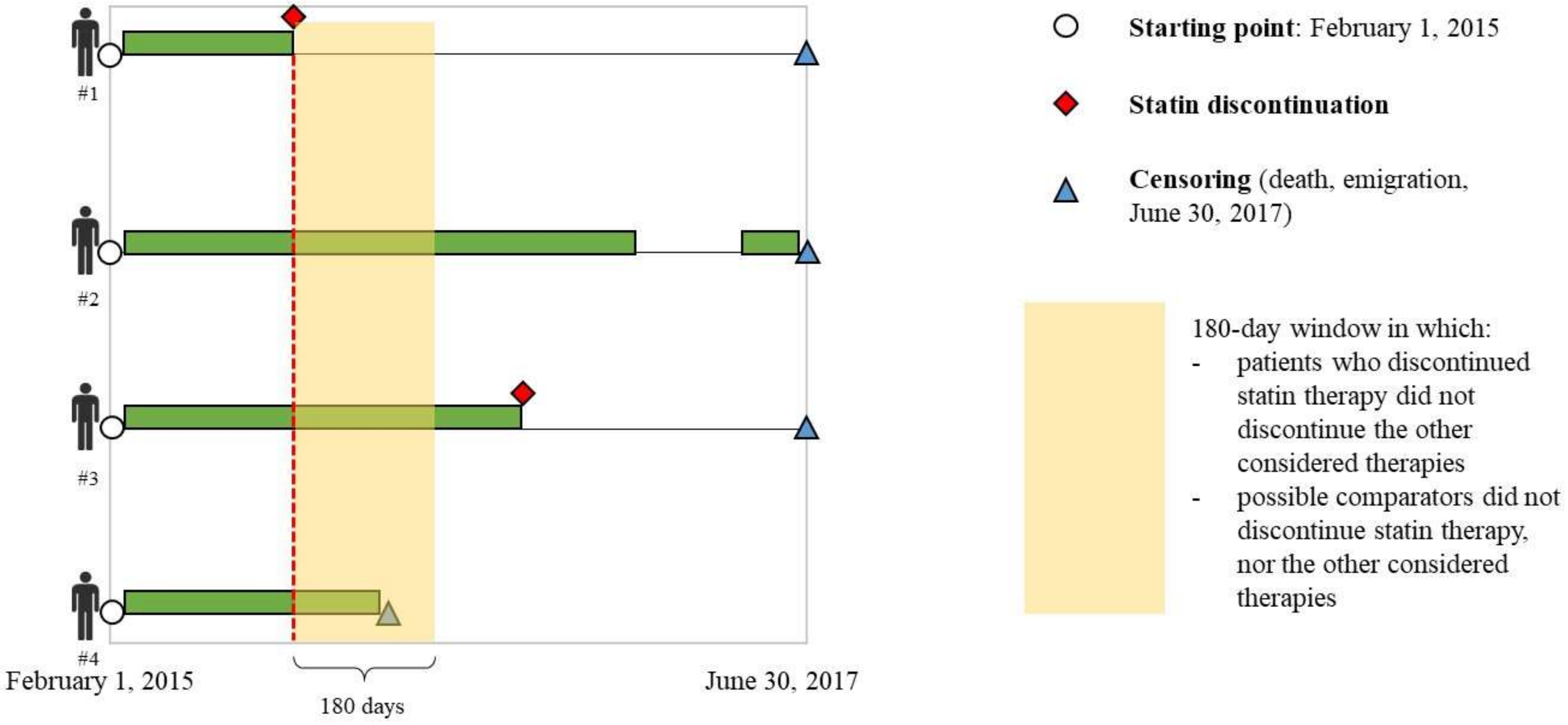

For each patient who discontinued statin therapy, a comparator was identified. To be included as a comparator, a patient did not have to discontinue the statin therapy at the discontinuation date of the corresponding patient and in the following 180 days. For example, patients #2, 3 and 4 are still on treatment with statins when patient #1 discontinued. Although patient #3 interrupted the drug treatment afterward, he could be selected as the comparator of patient #1 (because the discontinuation happens after the 180-day window starting from the discontinuation date of patient #1).

© 2021 Rea F et al. *JAMA Network Open.*

Conversely, patient #4 is not a suitable comparator for patient #1 because he/she did not accumulate at least 180 days after the discontinuation date of patient #1.

Finally, patient #1 and its possible comparators (patient #2 and 3) must not have discontinued the other considered drug therapies (i.e., blood-pressure lowering, antidiabetic or antiplatelet agents) in the 180-day window to be included in the analysis.

© 2021 Rea F et al. *JAMA Network Open.*

eFigure 3. Representation of Step-2 Follow-up Aimed to Assess Clinical Consequence of Discontinuing Statins

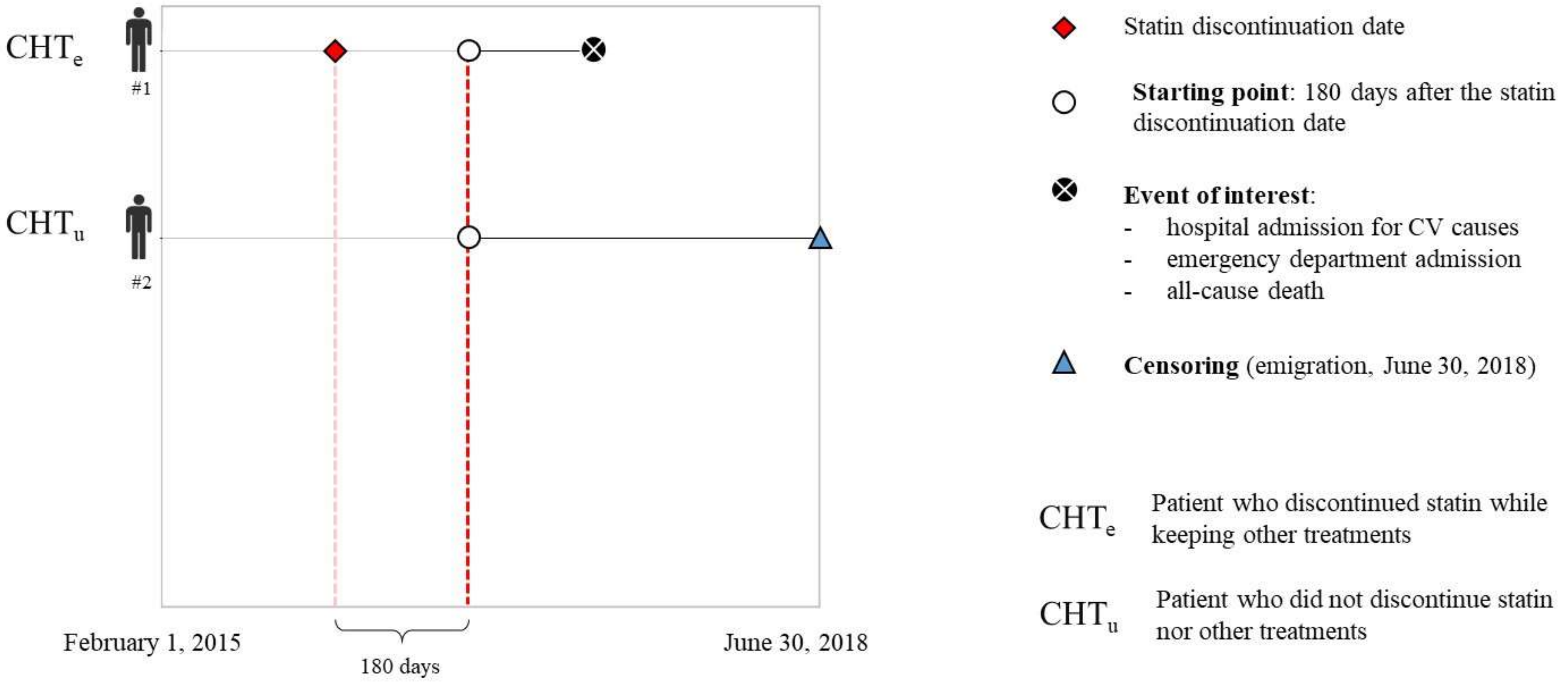

Assuming that patient #2 was randomly selected to be matched for propensity score for patient #1, both subjects were followed from 180 days after the discontinuation date of patient #1 until the occurrence of the clinical outcome of interest (i.e., hospital admission for CV causes, emergency department admission and all-cause death) or censoring (emigration or the end of data availability, i.e., June 30, 2018).

© 2021 Rea F et al. *JAMA Network Open.*
